# Redesign of the Geometry of Parts Produced from PBT Composite to Improve Their Operational Behavior

**DOI:** 10.3390/polym13152536

**Published:** 2021-07-31

**Authors:** Dan Dobrotă, Sergiu Viorel Lazăr

**Affiliations:** 1 Faculty of Engineering, Lucian Blaga University of Sibiu, 550024 Sibiu, Romania; 2 S.C. Contintental Romania, 550018 Sibiu, Romania; sergiu.lazar@ulbsibiu.ro

**Keywords:** PBT-GF30 (70% polybutylene terephthalate +30% fiberglass), composite central design, injection molding, artificial aging, viscosity

## Abstract

Parts produced from PBT-GF30 (70% polybutylene terephthalate +30% fiberglass) are very often used in car construction, due to the properties of this material. The current trend is to make parts with a shape designed to be as complex as possible, to take over many functions in operation. During the research, a part that is a component of the structure of car safety systems, and that must be completely reliable in operation, was analyzed. This piece has a complex shape that involves the intersection of several walls. Thus, the research aimed at establishing the optimal radius of connection between the walls (R), the ratio between the thickness of the intersecting walls (K) and the angle of inclination of the walls (α). The composite central design method was used to design the experiments. Both new parts and parts subject to an artificial aging process were tested. All parts were subjected to shear stress, to determine the load (L) and displacement (D) at which they break. In order to observe other changes in the properties of the parts, in addition to the mechanical ones, an analysis of the color of the new and aged parts was performed, as well as a topography of the surface layer in the breaking area. The design of the parts involved changes to the parameters of the injection process. In these conditions, a PBT-GF30 viscosity analysis was performed for new and artificially aged parts.

## 1. Introduction

The combination of high mechanical and electrical properties, good thermal stability and superior chemical resistance creates many opportunities for automotive applications for both PBT and PBT matrix composites, such as PBT-GF30. This composite offers good durability in conditions of heat stress and/or in harsh chemical environments, as found around certain parts used in the automotive industry. Thus, this material is most commonly used in the exterior and safety applications of the car body, including airbag covers, brake and duct clamps, cable gaskets and energy distribution boxes. In recent decades, high-performance plastics such as PBT-GF30 have experienced a boom in the production of various parts in the structure of cars. It is sometimes considered that the use of polymeric materials allows more freedom in the design of parts, but still, some problems have not been completely solved in this regard [1,2,3].

Because PBT-GF30 is used to make parts with complex geometric configurations, a series of principles must be taken into account when designing the shape of each part: the effect of the thermal node increases as the angles of the edges are sharper [4]; wall intersections must be produced in such a way as to avoid excessive wall thickening and the formation of thermal nodes; the thickness of the wall of the piece must be as uniform as possible, the ratio of the sections must not be too high, the passages between the walls of the piece must be produced progressively, without sudden openings [5]; it is advisable to design certain inclinations of the part walls in order to facilitate the removal of the piece from its mold or die [6].

One of the most important parameters to consider when creating parts from PBT-GF30 refers to the viscosity of the material during injection molding. This parameter is highly dependent on temperature and shear rate [4]. 

A possible technical solution for obtaining parts with the appropriate properties is represented by water-assisted injection molding technology, which has proven to be an important breakthrough in the manufacture of plastic parts [2,6]. The water-assisted injection molding process can also allow for greater design freedom, material savings, weight reduction and cost savings [7,8]. Although the elimination of water after injection is a problem in the case of this technology, it is applied on a very large scale; thus, the designing of the shape of the parts represents a solution for achieving very good injection molding (from the points of view of temperatures and the pressure of injection) and to obtain parts with good performance.

Another possible technological solution for obtaining PBT-GF30 parts with a complex geometry refers to the use of selective laser sintering (SLS) technology; however, in this situation, there is a risk of thermal degradation of the material, with negative effects on the behavior of the parts in operation [9,10,11]. 

In addition, the parts produced by SLS showed inferior mechanical properties to those cast by injection and, thus, this type of technology is not indicated for use for complex parts subject to heavy mechanical stress in operation [12,13,14]. 

The choice of a polymeric material for structural purposes, as is quite often the case with PBT-GF30, requires that the properties of the material be correlated with structural requirements and the influence of external factors (especially when in operation), taking into account the behavior of the material at high and low temperatures, aggressive environments, the influence of humidity (which especially affects polyamides), the type and duration of the load, and the rate of deformation. All this can be influenced to a very large extent by the constructive shape of the parts [15,16,17].

The design of PBT-GF30 parts and components requires the implementation of knowledge from different areas of engineering. For this reason, a successful design process requires both a knowledge of tool design and parts-manufacturing technologies. Thus, for a quality PBT-GF30 part, the following factors must be taken into account: design, processing, machining, handling and assembly, and life expectancy [18,19,20].

The improper design of parts produced from PBT-GF30 can lead to cracking trends during the manufacturing process or after a short period of use [21]. Thus, depending on the design of the products, manufacturing imperfections, as well as a decrease in cracking tendency during their use, can be avoided [22]. The design of parts produced from PBT-GF30 can also affect the microstructure of these composites, and significant segregation of the fillers can occur. This possible segregation can substantially affect the operating behavior of parts produced from such materials. Thus, it is necessary to control several parameters in the design process of products that may have a direct impact on the microstructure of the material, to ensure that the mechanical properties and dimensional accuracy correspond to their applications in the automotive industry [23]. 

Achieving a correct design for parts produced from PBT also allows a reduction in the fragility of the parts. Thus, the size of the PBT granules influences both the manufacturing technology and the design of the parts produced from this material. It has been shown that choosing a part design based on the size of the PBT granules can determine the behavior of the part when in use [24]. As a result, for a grain radius of up to 46 μm, the ductile behavior of the parts can be improved, while for larger radii, brittle behavior can occur [25]. 

The process of obtaining PBT-GF30 parts by injection molding is a very complex one and determines changes in the thermomechanical properties of the materials, due to the very varied loads to which they are subjected at different stages of the technological process. Thus, at the beginning of the manufacturing process, all factors that may affect injection molding must be analyzed before deciding on the feasibility of manufacturing a product that has a certain complexity in its geometric shape [26,27]. 

The factors to be taken into account when manufacturing a PBT-GF30 product can be grouped into machine parameters (independent variables), process parameters (dependent variables) and quality indicators (final answers), comprising mainly the shape and dimensions of the part (product design) [28,29]. At present, a number of algorithms can be used to design the shapes of parts, such as the genetic algorithm (GA). Thus, using an intelligent GA-based system, a new conceptual design system can be generated that avoids human errors and eliminates part design defects [30,31,32,33].

Considering these matters, the research carried out in this paper aimed to ensure product quality indicators by adopting an appropriate design. From the analysis of the current state of research, it can be concluded that the design of an optimal geometry for the parts in PBT-GF30 can contribute to a substantial improvement in their performance once in operation. Thus, the main objective of the research presented in this paper is represented by the optimization of the construction of parts to ensure their successful operation throughout their life. In this sense, the research aimed at optimizing the constructive form using a central composite design method. Thus, the aim was to determine the optimal shape of the parts, taking into account the forces, i.e., the displacements, by which the breaking of these new parts occurs, as well as in those that have been subjected to an artificial aging process. Due to the fact that the design of the parts involves changes to the process of obtaining the parts by injection molding, an analysis of the viscosity of PBT-GF30 from both the new and artificially aged parts was performed. At the same time, we analyzed how the topography of the parts’ material layer changes in the breaking area. The results obtained in the experimental research were also processed using an analysis of variance (ANOVA). The rest of the paper is structured in the following sections: Materials and Methods, Results and Discussions, and Conclusions.

## 2. Materials and Methods

### 2.1. Materials

The operating behavior of the parts produced from PBT-GF30 depends very much on their geometry. This is especially true for parts where the geometric shape involves sudden changes in wall size. This type of part presents high risks in operation, given that the sudden transition from one section to another is not achieved through surfaces with certain geometric characteristics. For this reason, during the research, a part used in the automotive industry was analyzed that has large variations in its sections.

#### Properties of PBT-GF30 Material

The part considered in the research was of the housing type, being produced from PBT-GF30 material (70% polybutylene terephthalate +30% fiberglass). This material was produced by Shanghai Langqi Plastic Materials Co., Shanghai, China, under the trade name DSM Arnite^®^ TV4 261. This part was obtained by injection molding, and the following technological parameters were taken into account: mold temperature (85 °C); melt temperature (223 °C), injection pressure (125 MPa); residence time (6.5 min). 

PBT-GF30 is a thermoplastic, semi-crystalline plastic of the polyester family, which crystallizes very slowly and is, therefore, in an amorphous-transparent or crystalline-opaque state, depending on the processing method. It is distinguished by its high strength, rigidity and dimensional stability under heat, as well as by its very high dimensional stability and low creep. In addition, PBT exhibits, like polyesters in general, very good friction and wear properties. Unlike PET, PBT has better impact resistance, especially in cold temperatures. The properties of PBT-GF30 are optimized in different areas compared to PBT. The properties of PBT-GF30 are as follows: high strength and rigidity; dimensional stability; low creep; very good friction and wear resistance; good impact resistance; very low thermal expansion; good chemical resistance to acids; very good electrical properties; very low water absorption; and being easy to bond and weld. 

Regarding the distribution of the fiber length in such a composite material, this can be established by considering the weighted average fiber length (*L_w_*):

(1) $$L_{w}=\frac{\sum_{i=1}^{n}n_{i}\times L_{i}^{2}}{\sum_{i=1}^{n}n_{i}\times L_{i}}$$

It was found that the weighted average fiber length (*L_w_*) for the raw material equals 327 μm, with an average length of 254 μm and a median length of 233 μm. The properties of the PBT-GF30 composite are presented in Table 1.

**Figure 1:**
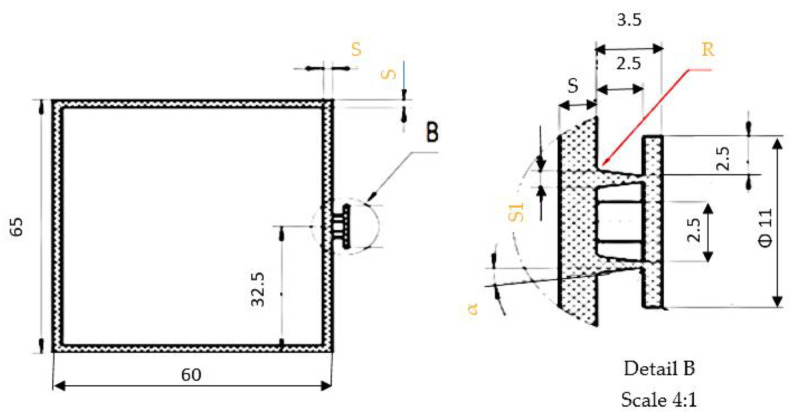
Shape and dimensions of the housing part. Optimized geometric parameters: R-radius of connection between the walls of the part; α—the angle of inclination of the part’s walls; K = S1/S—the ratio between the thickness of the part’s walls.

From the PBT-GF30 material, we produced a carcass-type part that had large variations in section and that had to behave very well under shear stresses. This type of stress was considered because it was applied to the part during use, at a force that caused shear stresses in the material from which it was produced. A sketch of this type of part is shown in Figure 1.

### 2.2. Methods

#### 2.2.1. Parts Testing Method

**Figure 2:**
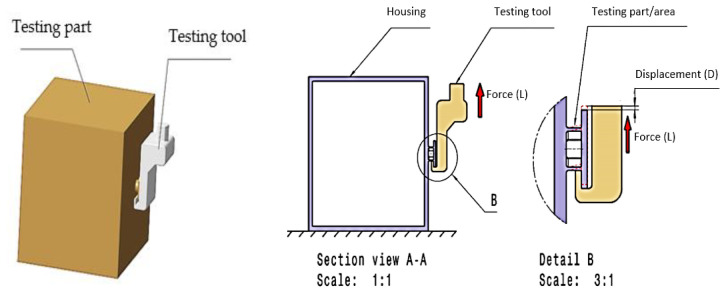
Loading scheme of housing parts.

The produced parts were subjected to a load, according to the sketch shown in Figure 2. Thus, a device was designed that allowed the proper testing of the parts, the shape of which is shown in Figure 2.

**Figure 3:**
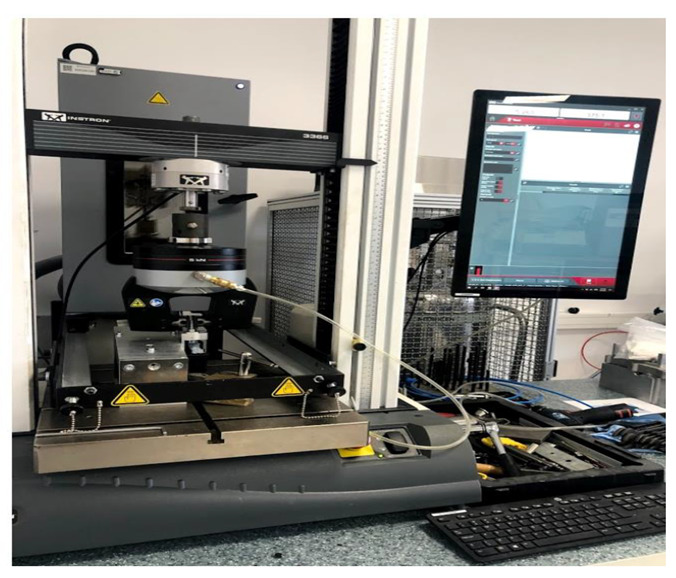
Setup of the testing machine, Instron 6800 Dual Column Table 68TM-30.

The tests were performed using an Instron 6800 Dual Column Table 68TM-30 test machine provided by Instron Schenck Technologie-und Industriepark, Landwehrstraße 65, D-64293 Darmstadt, Germany (see Figure 3). The power cell was 1 kN, so that the values are within the tolerance field.

The tests were performed at a temperature of 20 °C, by going through the following steps:

- The housing part was immobilized on the testing machine support;
- The device used for testing was calibrated and mounted;
- The parts were loaded, with a speed set at 35 mm/min.

The research aimed to establish the following parameters for each piece: displacement (mm) and force (N). It should be noted that those values of the parameters at which the parts broke were recorded.

#### 2.2.2. Artificial Aging of Parts

A thermal chamber was used to perform the artificial aging of the parts—a Temp Shock test chamber Votsch VT3 7012 S2 produced by the Test Equipment Co., Ltd., Jin Hui Industrial Park, Yixing, China. This thermal chamber allows very fast temperature changes in the range of −50 °C to +140 °C. This way of testing this type of part permits a reduction in the incidence of early failures and the improvement of their manufacturing conditions, to increase their reliability in operation.

As regards the artificial aging regime, it has been established by taking into account the following parameters:

- The parts were kept in the thermal chamber for 1250 h, at temperatures in the range of −50 °C to +140 °C;
- It was maintained for one hour at −50 °C, and for the next hour at +140 °C;
- The humidity varied between 60% and 95%.

These parameters of the aging process were established by taking into account the fact that the part is within the structure of a car, and the conditions of the part when in use correspond to those regulated in the research. It should also be noted that these aging conditions correspond to a duration of 15 years’ use of the car part.

#### 2.2.3. Checking the Geometry of the Parts

**Figure 4:**
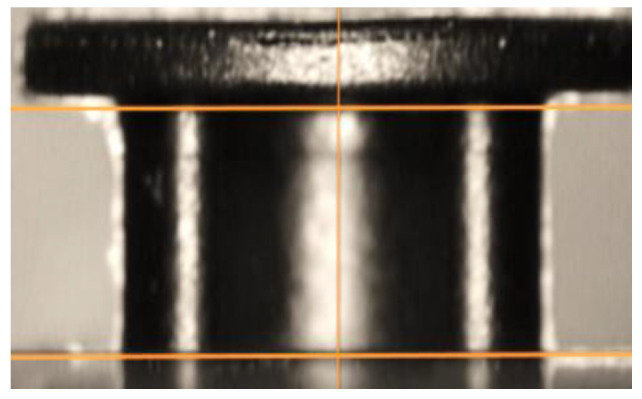
Checking the dimensions and geometry of the parts.

Due to the fact that the analyzed parts have a rather complex shape, before being tested, they were subjected to a measurement process (see Figure 4). This measurement process had to be performed because the research aimed at optimizing the geometry of this type of part to behave better in operation. A CRYSTA-Apex V measuring machine, produced by Mitutoyo, Japan, was used to measure the geometry of the parts; this device is able to perform very precise measurements at high speed. We used this type of machine because it has a high measuring accuracy and allows temperature compensation from 16 °C to 26 °C. Following the verification of the dimensions and geometry of the parts, only those parts that corresponded to the geometry imposed by the research methodology were subjected to testing. 

#### 2.2.4. Scanning Electron Microscopy (SEM) Analysis of the Sampled Surfaces in the Breaking Areas

Important information on how the tested parts behave can be obtained by microscopic analysis of the surfaces of the parts in the breaking area. For this reason, SEM analysis was performed, both for the breaking surface of the new part and for the part subjected to the artificial aging process. Thus, information was obtained about the topography of the breaking surface for the two types of tested parts. An AIS2100C electron microscope produced by Seron Technologies, Inc., 5F World Vision Bldg., 209, Gyeongsu-daero, Uiwang-si, Gyeonggi-d, Korea was used in the research. 

#### 2.2.5. PBT-GF30 Viscosity Analysis

The design of the parts influences the parameters of the technology for obtaining the parts by injection molding. Thus, the correct design of the parts, and the establishment of correct parameters for the technology of obtaining the parts, can determine a decrease in the degree of degradation of the viscosity for PBT-GF30. Under these conditions, during the research, the aim was to determine the viscosity of PBT-GF30 in the form of new materials, both materials from the new parts and from those subject to the aging process. Viscosity measurements were performed in accordance with DIN EN ISO 1628-5. 

To obtain the polymer solution, the solvent 1,2 dichlorobenzol-phenol 1:1 was used. The polymer solution, with a concentration of 0.0047 g/cm^3^, was analyzed using an Ubbelohde viscosimetric device. To solubilize the materials, the mixtures containing the polymer and solvent were heated, while being stirred, at 135 °C for 2 h. The solution was then cooled and poured into an Ubbelohde viscometer tube, placed in a thermostatic water bath at 24 °C. The viscosity number (also referred to as “reduced viscosity *η*_red_ in cm^3^/g”) of the polymer was calculated using the solvent flow time and the flow time of the polymer solution in the viscometer, using Equation (2):

(2) $$\eta_{\mathrm{red}}=\frac{t-t_{0}}{t_{0}\times C}$$

where *t* is the flow time in seconds, *t*_0_ is the reference flow time of the solvent, and *C* is the concentration of the sample (g/cm^3^).

#### 2.2.6. Establishing the Correlation between the Design of the Part and the Modeling of the Injection Molding Process (IM)

**Figure 5:**
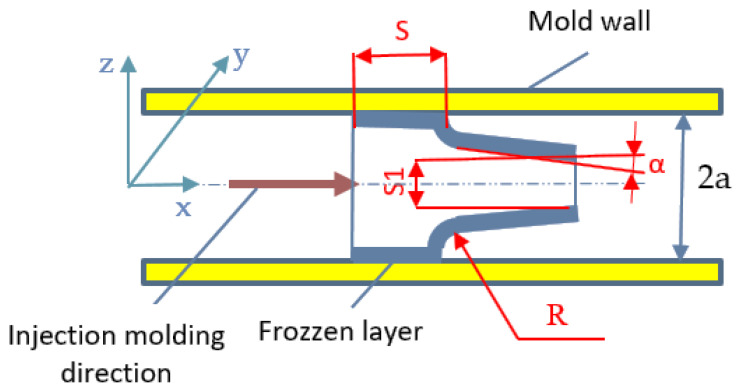
Cross-sectional view of the flow front in the Hele–Shaw model.

During the injection molding process, it is necessary that the filling of the mold takes place in good conditions, and this is influenced by the design of the parts. For this reason, it was necessary to find the mathematical model for the filling stage that best corresponded to the conditions imposed by the type of part analyzed. From the analysis of the shape and dimensions of the piece, it was found that the piece had walls with quite small thicknesses and, thus, the mathematical model that was indicated for use was the generalized Hele–Shaw model [34]. This is used for shear flow analysis in a thin cavity (two-dimensional formulation (2D), and considers the polymer to be an incompressible, generalized, non-Newtonian fluid under nonisothermal conditions. When using this generalized model, it is assumed that the width of the space between two plates is much smaller than the other dimensions of the polymer flow (see Figure 5).

Due to the fact that the research required the optimization of the three parameters, R, K and α, it follows that the half-thickness of the core feedstock, *δ*, is variable in time (*t*), depending on the values of the three parameters; however, the three parameters are also variable on the *x* and *y* directions. The momentum equation for the filling phase of the injection molding process, assuming the Hele–Shaw simplification, is expressed as:

(3) δ=δ(x, y, t), δ=δ(R, K, α, t)

According to the basic equation of fluid mechanics [34,35], the continuum model of the injection molding process is expressed as:

(4) $$\frac{\partial }{\partial x}\left(S\frac{\partial p}{\partial x}\right)+\frac{\partial }{\partial y}\left(S\frac{\partial p}{\partial y}\right)=0$$

where *p* is the pressure of the feedstock and *S* is the flow conductance, which is calculated as:

(5) $$S=\int_{0}^{\delta }\frac{z^{2}}{\eta_{c}}dz+\int_{\delta }^{b}\frac{z^{2}}{\eta_{s}}dz$$

where *b* is half of the thickness of the core feedstock, *η_c_* and *η_s_* are the viscosities of the core feedstock and the skin feedstock, respectively, and *δ* is half of the plate thickness. The energy equation is expressed as:

(6) $$\rho c_{p}\left(\frac{\partial T}{\partial t}+u\frac{\partial T}{\partial x}+v\frac{\partial T}{\partial x}\right)=\lambda \frac{\partial^{2}T}{\partial z^{2}}+\eta {\dot{\gamma }}^{2}$$

where ρ, *c_p_*, *λ* and $\dot{\gamma }$ are the density, specific heat capacity, thermal conductivity and shear rate of the feedstock, respectively; *u* and *v* are the velocities in the *x* and *y* directions.

The determination of the shear rate of the feedstock ($\dot{\gamma }$ ) is made using Equation (7):

(7) $$\dot{\gamma }=\sqrt{{\left(\frac{\partial u}{\partial z}\right)}^{2}+{\left(\frac{\partial v}{\partial z}\right)}^{2}}$$

In addition, the boundary conditions for the generalized mathematical model, according to Fernandes et al. [34], are given by Equations (8)–(10):

(8) $$u=v=0,\;T=T_{W}\;at\;z=a$$

(9) $$\frac{\partial u}{\partial z}=0;\;\frac{\partial v}{\partial z}=0;\;\frac{\partial T}{\partial z}=0,\;at\;z=0$$

(10) p=0 along the flow front

All these boundary conditions cannot be fully applicable to research related to the optimization of the design of parts produced from PBT-GF30, because changing the geometry of the parts, given the three parameters R, K and α, can determine a variation of the flow rate of the polymer along the *z*-axis. By a modification of the geometry of the parts, the flow section of the material changes, and this influences the size of the flow along the *z*-axis. Thus, the conditions at the border can no longer be used, as happens in the scenario of the ideal flow. However, this mathematical model can be used to interpret the experimental results obtained in terms of forces and displacements that occur when breaking parts are produced with different values for R, K and α.

#### 2.2.7. Research Methodology

In the experimental research, the design of the experiments conducted by the central composite design method, based on the response surface methodology, was considered. This method also involves the use of central and axial (or stellar) points and allows the estimation of second-order effects.

Adding axial points practically increases the number of levels to five. This can cause problems if the axial points cannot be rolled for technical or safety reasons. For a design with *n* factors, the distance of the axial point from the design center is α = 2^*n*/4^. The research, based on input factors and their levels, involved 20 sets of experiments. The design of the experiments was performed using the statistical software MINITAB. Given that the number of the input factor was *n* = 3, the value of α was set at a value of 1.68179. The natural and coded levels of the independent variables for the design of the experiments are presented in Table 2.

After defining the levels of the cutting parameters, the sequences of experiments were generated using MINITAB statistical software using central composite design. Table 3 presents the 20 sets of experiments in terms of the coded values of the parameters, according to the running order. The number of experiments was generated, based on the number of input factors and their levels.

The experimental research aimed to determine the values of force (N) and displacement (mm) recorded at the time the samples broke. These values were determined for those parts in a new condition and those parts that underwent an aging process.

## 3. Results and Discussion

The main purpose of the research was to determine the optimal geometry of the parts produced from PBT-GF30, to allow the best performance in their operation. Thus, the aim was to determine the behavior of the new parts as well as of those subjected to an aging process, at the point of shear stress. In this sense, second-order models were determined for both force and displacement. Following the determination of the mathematical models, the ANOVA analysis was used to establish which terms were significant and which were insignificant in the mathematical models. Insignificant terms were identified and removed. Thus, it was considered that the variable for which the value of *p* was less than 0.05 indicated that the term in the model had a significant effect on the response. In addition, after completing these stages, the mathematical models that were developed for strength and for displacement (D) were performed for both new parts and those subject to the aging process.

### 3.1. Results Obtained When Testing New Parts

After testing the new parts at the shear stress, the results presented in Table 4 were obtained. These results were established by taking into account both the force (L) and the displacement levels at which the parts broke.

Following the experimental research, the results were statistically processed, and the values obtained from the ANOVA analysis are presented in Table 5.

Following the processing of the experimental results, mathematical models were established that offer the dependence of the force (L) on the displacement (D) of the three parameters R, K, and α. Higher values for “F” and lower values of *p* (*p* < 0.05) indicate that the corresponding variable is very significant. Therefore, the results in Table 5 indicate that the influence of the parameters R × α, R × K, R × K × α, α^2^, and K^2^ is insignificant in the case of force (L). Consequently, these terms were removed from the complete model, and the mathematical model given by Equation (11) was obtained:

(11) $$L=184.688+62.865\times \;R+40.013\times K+31.411\times \alpha -6.241\cdot K\times \alpha -61.457\times R^{2}$$

In addition, from the analysis of the results presented in Table 5, it was observed that the influence of the parameters R × α, R × K, R × K·α, α^2^, and R^2^ is insignificant in the case of displacement (D); therefore, these terms were removed from the complete model, and the mathematical model given by Equation (12) was obtained:

(12) $$D=3.364+0.225\times \;R\;-\;2.022\;\times \;K\;-\;0.032\;\times \;\alpha \;+0.143\times \alpha \cdot K+1.374\times K^{2}$$

Thus, in the case of force (L), the mathematical model is extremely significant and is characterized by a correlation coefficient (R^2^) of 98.27%, which means that over 98% of the data can be explained by the mathematical model established using Equation (11). Additionally, in terms of displacement (D), the determined mathematical model is extremely significant and is characterized by a correlation coefficient (R^2^) of 93.31%, which means that over 90% of the data can be explained by the mathematical model established using Equation (12). In order to verify that the determined models are adequate, five validation experiments were performed, according to the data presented in Table 6. Other values were chosen for the three parameters than those used in the experimental research program, which fell between the minimum and maximum values of the previously defined levels. The values predicted using the mathematical models for force (L) and displacement (D) and the actual experimental values were compared. Percentage errors were calculated. All these values are presented in Table 6.

**Figure 6:**
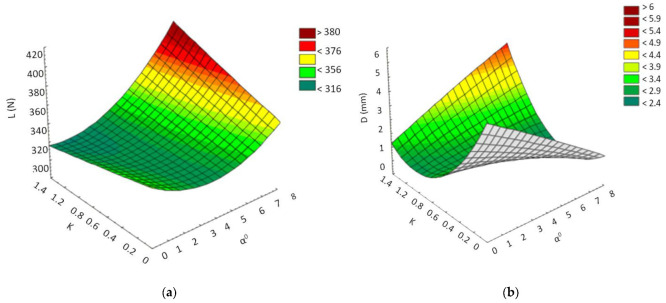
Graphs of the response area of the force of K and α° (**a**) and the displacement D, as a function of α° and K (**b**).

The percentage error between the actual and estimated value ranges for force is from 1.966 to 5.095%, i.e., technically acceptable. In addition, in the case of displacement, the percentage error interval between the real and the estimated values is between −5.977% and 8.101%, which demonstrates that the use of the mathematical model instead of experimental research allows the obtaining of predicted values that are very close to the real values. From the confirmation experiments, it was observed that the developed models can predict with very good accuracy both the force (L) and the displacement (D) levels. In addition, from the analysis of the values of F presented in Table 4, it was observed that the interaction between the angle of inclination of the walls of part α and the ratio between the thicknesses of the walls of part K has the greatest influence on the values of force and displacement at which the rupture occurs. Thus, the graphs of the response surface of the force and the displacement were produced according to the two parameters (see Figure 6).

From the analysis of the graphs presented in Figure 6, it can be observed that, if we consider only the two parameters α° and K, we can obtain a high value of the force at which the part breaks, for the situation in which the two parameters have high values. However, supraunitary values for K would cause a thickening of the part walls, which could lead to areas with inadequate properties arranged at the intersections of the part walls. Under these conditions, it can be appreciated that corresponding values for the force (L) and displacement (D) can be obtained for values of K = 0.8–1.0 and angles of α = 6–8°, respectively. In addition, according to those values presented in Table 5, considering the values of F, the greatest influence on the force at which the part break occurs has an angle α of inclination of the walls and the connecting radius R between the walls of the part. Thus, for a correct assessment of the optimal values of the three parameters for which the force has a maximum value and the displacement is at a minimum, this can be achieved only in those conditions in which their cumulative influence is taken into account.

Under these conditions, based on the experimental results that were obtained and are presented in Table 4, it was observed that the maximum value for a breaking force L_max_ = 378.8 N and the minimum displacement D_min_ = 2.986 mm are obtained for the following values of the parameters, R = 1.104 mm; α = 6°, K = 1, respectively. Thus, it can be seen that the cumulative analysis of the three parameters obtains approximately the same optimal values as are achieved when only the parameters α and K are taken into account. In addition, the application of the experimental design through *central composite design* allowed the establishment of an optimal value for the connection radius R that could not have been taken into account if other methods of designing the experiments had been applied.

In addition, according to Table 4, the percentage difference between the maximum and minimum values of the force at which the parts broke is 29.45%, which demonstrates that a correct design of the shape of the parts causes a substantial increase in breaking force. In the case of displacement (D), it was observed that a change in the geometry of the part causes a fairly large variation in its value, in the sense that the percentage difference between the minimum and maximum value is 42.50%. This demonstrates that, by adopting an optimal geometry of the parts, it is possible to obtain an increase in the performance of the parts when in operation.

The experimental results, as well as their statistical processing, have shown that a connection radius between the walls of the parts that is as large as possible can cause a reduction in certain tendencies to crack the material. In addition, an appropriate angle of inclination or an optimal ratio between the thicknesses of the walls determines a reduction in the agglomerations of material that can cause inhomogeneities of the structure [36,37].

The adoption of a certain design of the parts can determine the appearance of certain inflection points, in terms of the evolution of temperature and pressure during the injection molding process. Thus, temperature differences cause the crystallization of the polymer at different intervals, and detachment of the polymer from the surface of the mold cavity may occur in certain areas, due to its increasing density [38,39]. In these conditions, it is confirmed that the use of a connection radius R between the walls of a very large part determines a large variation of temperature, due to the agglomerations of material that may occur. In addition, the research that has been carried out contributes to a great extent to finding solutions to optimize the design of the parts, so that there are no high inflection points for temperatures that cause different kinetics of crystallization in the *z*-axis (Figure 5). At the same time, the adoption of a high K ratio can cause temperature inflection points, with negative effects on the polymer crystallization process. Under these conditions, research has confirmed that, for K = 1, the best parts are obtained in terms of breaking behavior, because the temperature inflection points are avoided and uniform crystallization of the polymer is obtained.

From the analysis of Equation (6) in the generalized Hele–Shaw model, it was observed that the shear rate depends on the flow rates of the polymers *u* and *v* along the x and y axes, respectively (Figure 5). These flow rates of the polymer may undergo certain changes in values due to changes in the design of the parts, with an effect on the shear rate and, thus, on the mechanical properties of the material in the part. For this reason, these parameters can be important factors that contribute to the phase-change process of the polymer. Under these conditions, it is confirmed that it is necessary, when designing the shape of the parts of the dynamics, to take into account the polymer plasticization process that takes place during the technological process of injection molding [40,41]. As a result, the choice of small values for the R-ray would cause an increase in the flow rate of the polymer, which can cause substantial changes in the values of the shear rate $\dot{\gamma \;}$(Equation (7)), with effects on the mechanical properties of the polymer in the parts.

### 3.2. Results Obtained When Testing Artificially Aged Parts

In order to be able to observe the behavior of the parts under real operating conditions, they underwent an artificial aging process, as presented in Section 2.2.2. These aging conditions of the parts correspond to the conditions in which the car, and the structure in which the part is included, are used for a period of 15 years. The testing of artificially aged parts was performed under the same conditions as in the case of new parts. Thus, following the testing of artificially aged parts at the shear stress, the results presented in Table 7 were obtained. These results were established, taking into account both the force (L) and the displacement (D) at which breaking of the parts occurred.

The results obtained in the experimental research were statistically processed, and the values obtained from the ANOVA analysis are presented in Table 8.

By processing the results obtained in the experimental research, two mathematical models were established that offer the dependence of force (L) and displacement (D), depending on the three parameters considered, namely, R, K, and α. To determine whether a variable is significant or not, the values of “F” and *p* were considered. Thus, variables with values for *p* < 0.05 indicate that the corresponding variable is very significant, and the high values of “F” also indicate that the variable is significant. Therefore, the results in Table 5 indicate that the influence of the parameters R × K, R × K × α, R^2^, and K^2^ was insignificant in the case of force (L). Therefore, these terms were removed from the complete model and the mathematical model that was obtained is represented by Equation (13):

(13) $$F=65.073+206.2\;\times \;R-7.017\;\times \;\alpha +176.164\;\times \;k-72.808\;\times \;R\;\times \;\alpha -7.017\;\times \;k\;\times \;\alpha -2.044\;\times \;\alpha^{2}$$

From the analysis of the results presented in Table 8, it was also observed that the influence of the parameters R × K, R × K × α, R^2^, and α^2^ was insignificant in the case of displacement (D). As a result, these terms were removed from the complete model and the mathematical model given by Equation (14) was obtained:

(14) $$D=1.831-0.041\;\times \;\alpha +0.361\;\times \;R-1.315\;\times \;K+0.334\;\times \;\alpha \;\times \;K-0.195\;\times \;R\;\times \;\alpha +0.394\;\times \;K^{2}$$

The ANOVA results that were obtained showed that the mathematical models developed are extremely significant. Thus, in the case of force (L), the mathematical model is extremely significant and is characterized by a correlation coefficient (R^2^) of 96.83%, which means that over 96% of the data can be explained by the established mathematical model, using Equation (13). In addition, in the case of displacement (D), the determined mathematical model is extremely significant since it is characterized by a correlation coefficient (R^2^) of 97.28%, which means that over 97% of the data can be explained by the mathematical model established using Equation (14).

To verify that the determined models are adequate, five validation experiments were performed, according to the data presented in Table 9. Other values were chosen for the three parameters than those used in the experimental research program, which fall within the range of previously defined levels. The values predicted using the mathematical models for force (L) and displacement (D) and the actual experimental values were compared. Percentage errors were calculated. All these values are presented in Table 9.

The percentage error ratio between the actual and the estimated values for the force is from −5.906% to 1.638%, i.e., technically acceptable. In addition, in the case of displacement, the percentage error interval between the real and the estimated values is between −4.622% and 5.424%, which demonstrates that the use of the mathematical model instead of experimental research allowed us to obtain values very close to the real values.

**Figure 7:**
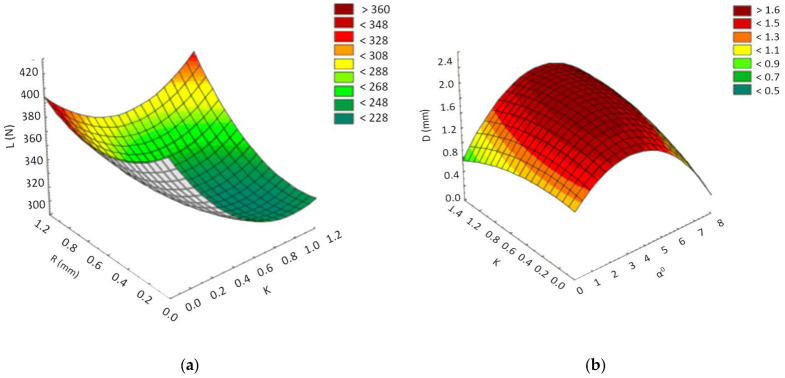
Graphs of the response surface for force (L) as a function of K and R (**a**), and for displacement (D) as a function of α and K (**b**).

From the confirmatory experiments, it was observed that the developed models can predict, with very good accuracy, both the force (L) and the displacement (D). From the analysis of the values of F presented in Table 8, it was also observed that the interaction between the connecting radius R and the ratio between the wall thickness of part K has the greatest influence on the force value, and in the case of displacement, the interaction between the inclination angle of the piece α and the ratio between the thicknesses of the walls of piece K have the greatest influence. Thus, the graphs of the response surface regarding force and displacement were produced, depending on the parameters whose interaction most influenced the force and displacement (see Figure 7).

The analysis of the graphs of the response surface (Figure 7) demonstrates that, in a situation where the interaction of R and K on L is considered, high values for force can be obtained if the two parameters take the highest values. Additionally, if we consider the interaction of K and α on D, we can obtain high values for displacement, for the situation where α = 4° and K = 0.8. Under these conditions, it can be appreciated that the choice of overly high values for K negatively influences the behavior of the parts subjected to the aging process. In addition, better preservation of the elasticity of the material in the part is possible if the angle α has average values of about 5°, in conditions where the parameter K is about 0.9.

Under these conditions, it can be appreciated that a thickening or thinning of the walls in the analyzed section of the part can cause faster aging of the material within it. One very important point is that which refers to the angle of inclination α, in the sense that too small an angle can cause an accumulation of stress in the material, and too large an angle causes a thinning of the part’s walls, with negative effects on the aging process. Additionally, if we consider separately the interaction of the three parameters on L, according to those presented in Table 9, given the values of F, the greatest influence on the force has the parameter K and the connection radius R, and the least influence, the angle α. Thus, it was demonstrated that an accumulation of material in the area of the intersection of the walls in the parts had a considerable influence on the magnitude of the force at which the rupture occurred.

Regarding displacement (D), given the separate interaction of the three parameters on it, according to the values presented in Table 8, it was observed that, given the values of F, the greatest influence on the displacement was the connecting radius R, specifically the parameter K, and the smallest influence, the angle α. Thus, it can be appreciated that the greatest influence on the aging process of the part’s material was the connection radius between the part walls (R), and the ratio between the wall thicknesses of the part (K).

Under these conditions, based on the experimental results, as presented in Table 8, it was observed that the maximum value for the breaking force L_max_ = 298.5 N is obtained for the following values of the parameters: R = 0.9 mm, α = 6°, and K = 1. Thus, it was found that the highest values for L were obtained for the parts subjected to the aging process in approximately the same conditions as the new parts, with the difference that the optimal radius value for the aged parts was lower than in the case of new parts. This can be explained by the fact that too great a connection radius can cause the material to thin in some areas and thicken in other areas, with negative effects on the aging process.

From the analysis of the results presented in Table 7, a percentage difference of 42.47% was observed between the maximum and minimum values of the force at which the parts broke, which is much higher than in the case of new parts (29.45%). This demonstrates that the adoption of optimal part geometry contributes to an increase in the performance of the parts during their use. In the case of displacement (D), it was observed that, in the case of parts subject to the aging process, the percentage difference between the minimum and maximum values was 49.54%, i.e., higher than that in the case of new parts, which was 42.50%. This demonstrates that, by adopting an optimal geometry of the parts, it is possible to obtain an increase in their operating performance, leading to an increase in their lifespan.

In addition, in the case of displacement (D), the minimum value D_min_ = 1.071 mm was obtained for the same values for which the maximum force was obtained, R = 0.9 mm; α= 6°, K = 1, respectively. This demonstrates that the adoption of these constructive parameters for the parts ensures good performance in their operation throughout their life period. Regarding the effect of the aging process on the characteristics of the material in the parts, it was found that a reduction of about 21% in the magnitude of the force at which the rupture occurred was obtained, while the reduction in displacement was much greater, about 50%. This confirms that the aging process has a much greater effect on the loss of elasticity of the material than on its breaking strength.

The experimental results, as well as their statistical processing, have shown that a connection radius between the walls of the parts that is as large as possible can cause a reduction in certain tendencies to crack the material. In addition, a correlation of the values of the three analyzed parameters (R, K, and α) can determine an increase in the lifespan of the parts [42,43].

### 3.3. Determination of the Evolution of PBT-GF30 Viscosity

The injection molding process of PBT-GF30 parts is influenced by many factors, and the design of complex thin-walled parts can influence a number of parameters, in particular, the shear stress and heat dissipation speed. These parameters can influence the viscosity of the part material, especially due to the occurrence of the phenomenon of chain scissions and polymer degradation. Under these conditions, the research undertaken took into account an analysis of the viscosity, and, implicitly, of the degree of degradation of the polymer for the new parts, specifically the artificially aged ones, depending on the design of the parts.

Thus, the viscosity was determined for all 20 types of parts with different designs, as presented in the research methodology. The granular material had reduced viscosity *η*_red_ = 99.7 (cm^3^/g). The results obtained after performing the viscosity tests are presented in Table 10.

The experimental results obtained at the stage of determining the viscosity of the material have shown that the design of the parts particularly contributes to the viscosity values. Thus, in the case of new parts, due to the degradation of the polymer bonds during the injection molding process, a degradation of the viscosity between 0.20% and 3.91% was obtained. This demonstrates that part design is a parameter that particularly influences the viscosity of new parts. Given that, for new parts, a maximum viscosity degradation of 4% is accepted, it results that a large part of them does not comply with this condition. In order to reduce the degree of viscosity degradation, it is necessary to optimize the other parameters of the injection molding process.

The injection molding process can cause a change in the average molar mass of the polymer, and this causes degradation of its viscosity [39]. This decrease in viscosity is mainly due to the fact that the design of the part influences the temperature reached by the polymer in different areas of the injection mold. This decrease could be considered, together with all other observations, as a consequence of the chain splitting phenomenon. Thus, PBT-GF30 can be subjected during injection molding to various degradation mechanisms that lead to cleavage of the polymeric bonds. In other words, the occurrence of a small number of scissions of chains induced by the manufacturing process could lead to a sharp decrease in viscosity and a decline in mechanical properties [44,45].

In the case of the parts subjected to the artificial aging process, the degradation process of the polymer is a much more accentuated one, showing degradation of viscosity of between 34.7% and 40.2%. This can be explained by the fact that the aging process of PBT-GF30 could also lead to crosslinking, by coupling the radicals held by the aromatic ring [46]; in other studies, this is explained based on the hypothesis of a possible crosslinking from the coupling between aliphatic radicals. In addition, the degradation of viscosity during the aging process of the parts confirms that PBT-GF30 mainly undergoes annealing rather than chemical crystallization during aging [47].

By increasing the temperature of the molten polymer and adopting an optimal geometry for the parts, a reduction of its viscosity can be achieved; this determines a smaller increase of the internal pressure during the filling process. High flow resistance of the polymer can be determined, mainly by the geometry of the part, not only by the parameters R, K, and α but also by the lower injection speed and the high viscosity.

The difference in viscosity of the plastic in each region of the mold cavity also affects the quality of the part. If appropriate values are not adopted for the three optimized parameters, local differences in viscosity for PBT-GF30 may occur in the intersection area of the part walls; therefore, the determination of the mechanical characteristics of the part is an important factor that allows us to understand the relationship between viscosity variation and injection molding conditions.

The mechanical characteristics of the part can be improved by changing the thickness of the part wall, and this is possible by adopting in the passage area between the part walls in the following domains of optimal values for the three parameters analyzed: K = 0.8–1.0; R = 0.6–0.8 mm, and α = 4–6°. Thus, it has been shown that viscosity is a very important parameter that affects the injection molding process of PBT-GF30 material, and this viscosity is also influenced by the microstructure of the material [48,49,50].

The design of the parts influences the size of the shear forces that may occur during the injection molding process. The size of the shear forces primarily causes a reduction in the molecular weight of the polymer, which has negative effects on the degradation of viscosity and on the mechanical properties of the material in the molded parts [51]. Adopting values that are too low for parameter K causes an increase in shear forces during the injection molding process. A reduction in the size of the shear forces can be obtained by choosing a connection radius between the walls of the part (R) that is as large as possible, but its value is limited when avoiding the occurrence of agglomerations of the material.

Based on the research conducted, it can be stated that the use of viscosity change assessment can be a criterion for establishing the links between the degradation of mechanical properties of parts and their design. In addition, the adoption of very low values for the angle α can lead to a low feed rate during the injection-molding process, and the PBT-GF30 material is subjected to a higher shear, which could lead to molecular degradation of the phase with a high concentration in PBT. Thus, the adoption of an improper design of the parts can lead to a decrease in viscosity values compared to mixtures obtained with higher feed rates. The reduction of viscosity is proof of the degradation of the mixture and a reduction of the mechanical properties of the parts (force—F, displacement—D). Therefore, a possible degradation of PBT chains would lead to a decrease in the size of their chains and the viscosity of the mixture. As a result, there may be an increase in crystallinity and a reduction in the impact of the properties [52].

Thus, the experimental results obtained demonstrate that there is a close link between the design of the parts produced from PBT-GF30 and the degradation of the viscosity obtained. It has also been shown that there is a correlation between the degree of viscosity degradation and the mechanical properties of the analyzed parts. Under these conditions, the research established a series of links between the design of the parts and the conditions to be considered during the injection-molding process, so as to obtain the best mechanical properties of the parts and the smallest degradations in the polymer viscosity.

### 3.4. Results Obtained When Measuring the Topography of the Parts’ Breaking Surface

The topography of the shape of the breaking surface of the parts is of particular importance because it can provide us with information on the way in which the breaking occurred, i.e., ductile breaking or brittle breaking. Ductile rupture is characterized by large deformations, and the rupture surface has a matte fibrous appearance [53]. The brittle fracture is characterized by small deformations and a shiny surface appearance. In this sense, the aim was to measure the surface roughness in the breaking area, but also to analyze the color, i.e., the brightness of the material. Thus, both for measuring the roughness of the breaking surfaces and for establishing the color and the brightness of the material, the methodology and equipment presented in Section 2.2.4 were taken into account. This analysis focused on the parts produced under the conditions where R = 0.9 mm, α = 6° and K = 1, respectively, because they had the best performance in the tests conducted on them.

**Figure 8:**
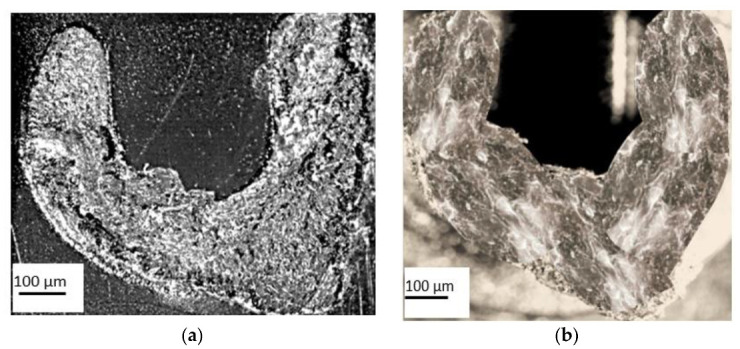
Macroscopic images of the part’s breaking area. (**a**)—new parts; (**b**)—parts subject to the process of artificial aging.

From the macroscopic analysis of the breaking area of the parts (Figure 8), it was found that, following the aging process, the material used to make them, PBT-GF30, changes color. This color change is more pronounced in the case of the matrix produced from PBT; thus, the aging process causes a transition from the black color of the composite matrix to a much lighter brown color. It was also found that, in the case of new parts, at the edge area of the broken material there was a surface specific to ductile rupture, and in the case of aged parts, the breaking surface was approximately uniform, as is specific to brittle fracture. Following the aging process, in addition to the change in the color of the matrix (PBT), it was observed that, in the breaking zone, glossy areas specific to the crystalline phase appear (Figure 8b).

These glossy areas are caused by a thickening of the crystalline phase induced by annealing and chemi-crystallization. These respective changes in crystallinity and fragility, obtained as a result of the aging process, are determined by the change in the molar mass of PBT. This means that the phase changes, along with the appearance of the crystalline phases, imply the appearance of fragility for the pieces produced from PBT [45].

The aging process to which the parts produced from PBT-GF30 were subjected determines an intensification of the depolymerization processes, as well as thermal destruction and thermo-oxidative degradation, which manifest as a reduction in the border viscous number and an increase in the number of carboxyl groups [46].

**Figure 9:**
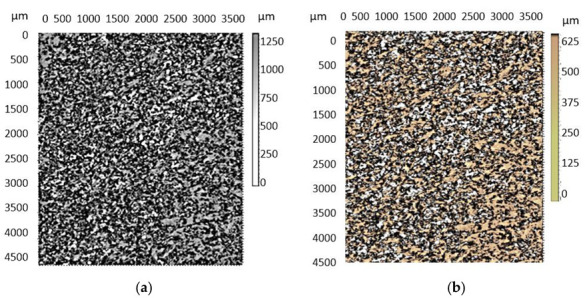
Topography of the surface layer of the part’s breaking surface. (**a**)—new parts; (**b**)—parts subject to the process of artificial aging.

In order to better observe the way in which the breaking surface changes the topography of its surface layer during the aging process, microscopic analysis was performed, and the obtained images are presented in Figure 9.

From the analysis of the images presented in Figure 8, it was observed that, in addition to a change in the color of the matrix in the PBT, there are also differences in the height of the roughness. Thus, in the case of new parts, a maximum roughness height of 1200 µm was measured, while in the case of parts subjected to the aging process, a maximum roughness height of 550 µm was measured. This difference in roughness height is determined by the fact that the type of rupture suffered by the tested parts changed from a ductile one, in the case of new parts, to a fragile one that was specific to the parts subjected to the aging process.

Under these conditions, it can be concluded that, depending on the design of the parts, changes can be obtained in terms of the mechanical strength, rigidity, microstructure and properties of the material. Therefore, fracture behavior investigation is a vital component of technical design [27,42].

## 4. Conclusions

The research carried out in this paper aimed to improve the characteristics of the parts made of PBT-GF30, by adopting an appropriate design. The optimization of the design of the parts must be correlated with the parameters of the technological process of injection molding. Thus, the modeling of the injection-molding process and the experimental results obtained showed that changing the values of optimized parameters (R—radius of connection between the walls of the part, α—the angle of inclination of the parts’ walls and K = S1/S—the ratio between the thickness of the parts’ walls) can cause a variation in the flow rate of the polymer and substantial changes in shear rate values (γ), with effects on the mechanical properties of the polymer in the molded parts. In addition, the analysis of the viscosity of the polymer in the particles, before injection molding, as well as that of the new parts and the artificially aged ones, showed that there is a close connection between the design of parts produced from PBT-GF30 and viscosity degradation.

Results obtained in the research showed that the design of parts produced from PBT-GF30, with respect to an optimal geometric shape, determines an increase in their operating performance. Thus, the analyses we performed showed the following:

- The best values for loading or displacement, in the case of new parts, were obtained if a connection radius was adopted between the parts’ walls of R = 1.104 mm, an angle of their inclination α = 6°, and a ratio between the thickness of the walls K = 1.
- The aging process of the material in the parts causes a partial change in the values of the parameters that were taken into the analysis. Thus, their optimal values are R = 0.9 mm, α = 6°, and K = 1. In this respect, it has been observed that, in order to improve the operating performance of the parts undergoing the aging process, a reduction in the value of the connecting radius is required; this is justified by the fact that too large a connecting radius can cause a thickening of the material in the area of intersection of the walls, with negative effects on the aging process.
- The characteristics of the material in the parts deteriorate with age and, in this respect, it was found that a reduction in the size of the load at which the rupture occurs was obtained by about 21%, while the reduction in the size of the displacement was greater than about 50%.
- Aging changes the color of the matrix (PBT) in the composition of PBT-GF30 from a black to a brown color. The topographic analysis of the breaking surface also showed that, in the case of the new parts, the maximum height of roughness was 1200 µm, while the roughness in the case of the parts subjected to the aging process was at a height of 550 µm.

The results obtained are very important safety information that can be used to make different types of parts from PBT-GF30, and, at the same time, these results are a basis for future research that considers other types of parts produced from different composite materials.

## Figures and Tables

**Table 1:** Properties of the PBT-GF30.

Bulk Modulus, GPa	Compressive Strength, MPa	Elastic Limit, MPa	Hardness, MPa	Tensile Strength, MPa	Modulus of Rupture, MPa	Breakdown Potential, MV/m
11.2	155	92	275	165	185	21

**Table 2:** Level parameters using the central composite design method.

Level	Lowest	Low	Center	High	Highest
Cod value	−1.68179	−1	0	1	1.68179
Connection radius, R, mm	0.096	0.3	0.6	0.9	1.104
Angle of inclination, α°	0.636	2	4	6	7.364
The report K = S1/S	0.195	0.4	0.7	1.0	1.204

**Table 3:** Sequence of experiments obtained using MINITAB.

Run Order	Connection Radius, R	The Angle of Inclination, α°	The Report K = S1/S
1	−1.00000	−1.00000	1.00000
2	1.00000	−1.00000	1.00000
3	1.00000	1.00000	−1.00000
4	0.00000	0.00000	1.68179
5	1.00000	1.00000	1.00000
6	1.00000	−1.00000	−1.00000
7	0.00000	0.00000	0.00000
8	0.00000	0.00000	0.00000
9	1.00000	1.00000	1.00000
10	1.00000	1.00000	1.00000
11	−1.68179	0.00000	0.00000
12	1.68179	0.00000	0.00000
13	1.00000	1.00000	1.00000
14	0.00000	0.00000	0.00000
15	0.00000	−1.68179	0.00000
16	0.00000	0.00000	0.00000
17	0.00000	0.00000	0.00000
18	0.00000	0.00000	0.00000
19	0.00000	0.00000	−1.68179
20	0.00000	1.68179	0.00000

**Table 4:** Force (L) and displacement (D) values obtained after testing new parts.

Run Order	R (mm)	α°	K	L (N)	D (mm)
1	0.300	2.000	1.000	314.4	2.504
2	0.900	2.000	1.000	298.7	2.763
3	0.900	6.000	0.400	357.7	3.219
4	0.600	4.000	1.205	327.4	3.702
5	0.900	6.000	1.000	359.7	2.919
6	0.900	2.000	0.400	292.7	3.389
7	0.600	4.000	0.700	322.3	3.007
8	0.600	4.000	0.700	326.4	3.101
9	0.900	6.000	1.000	341.8	2.991
10	0.900	6.000	1.000	345.7	2.839
11	1.104	6.000	1.000	378.8	2.186
12	0.096	6.000	1.000	339.3	3.651
13	0.900	6.000	1.000	355.5	3.802
14	0.600	4.000	0.700	323.9	3.005
15	0.600	0.636	0.700	357.7	3.501
16	0.600	4.000	0.700	335.4	2.614
17	0.600	4.000	0.700	327.5	2.998
18	0.600	4.000	0.700	322.7	2.563
19	0.600	4.000	0.195	328.8	3.733
20	0.600	7.364	0.700	335.6	2.267

**Table 5:** ANOVA analysis for force (L) and displacement (D), in response to the variations of parameters R, K, and α for new parts.

Source	Force (L)			Displacement (D)		
	Mean of Square	F Value	*p* Value Prob > F	Mean of Square	F Value	*p* Value Prob > F
Model	0.021	95.413	<0.001	0.058	107.669	<0.001
R	0.091	109.685	<0.001	0.035	136.669	<0.001
α	0.065	85.826	<0.001	0.069	99.749	<0.001
k	0.003	135.568	<0.001	0.001	115.1478	<0.001
R·α	4.57e−3	64.769	0.984	3.18e−3	83.616	0.627
R·K	2.31e−3	73.704	0.651	1.91e−3	77.750	0.322
K·α	3.19e−3	92.135	0.003	2.73e−3	95.994	0.001
R·K·α	4.13e−3	61.024	0.783	3.91e−3	67.523	0.211
R^2^	5.98e−3	105.136	0.002	3.95e−3	111.651	0.118
α^2^	1.43e−3	63.157	0.115	2.35e−3	105.637	0.189
K^2^	3.35e−3	71.125	0.236	1.57e−3	83.869	0.003
Residual	1.17e−3	1.123e−3	-	2.95e−3	1.318e−3	-

**Table 6:** Experiments performed to confirm the mathematical values for force (L) and displacement (D) for new parts.

No. Exp.	Parameters			Force (L)			Displacement (D)		
	R (mm)	α°	K	Predicted Value	Actual Value	% Error	Predicted Value	Actual Value	% Error
1	0.1	1.0	0.2	229.656	241.358	5.095	3.037	2.912	−5.977
2	0.4	2.0	0.4	276.235	281.667	1.966	2.755	2.621	−5.112
3	0.7	4.0	0.6	350.933	341.821	−2.596	2.658	2.763	3.800
4	1.0	5.0	0.8	350.197	367.742	5.010	2.622	2.854	8.101
5	1.1	6.0	1.1	374.639	383.372	2.331	2.572	2.495	−3.086

**Table 7:** Force (F) and displacement (D), obtained by testing artificially aged parts.

Run Order	R (mm)	α°	K	L (N)	D (mm)
1	0.300	2.000	1.000	211.9	1.229
2	0.900	2.000	1.000	202.3	1.439
3	0.900	6.000	0.400	245.3	1.719
4	0.600	4.000	1.205	207.4	2.127
5	0.900	6.000	1.000	255.1	1.537
6	0.900	2.000	0.400	283.7	2.008
7	0.600	4.000	0.700	219.8	1.991
8	0.600	4.000	0.700	207.5	1.987
9	0.900	6.000	1.000	291.2	1.781
10	0.900	6.000	1.000	209.4	1.431
11	1.104	6.000	1.000	251.3	1.372
12	0.096	6.000	1.000	217.2	1.163
13	0.900	6.000	1.000	298.5	1.071
14	0.600	4.000	0.700	217.8	1.287
15	0.600	0.636	0.700	239.7	1.219
16	0.600	4.000	0.700	222.9	1.879
17	0.600	4.000	0.700	219.2	1.106
18	0.600	4.000	0.700	223.9	1.198
19	0.600	4.000	0,195	287.1	1.098
20	0.600	7.364	0.700	211.8	1.169

**Table 8:** ANOVA analysis for force (L) and displacement (D), in response to the variation of parameters R, K, and α, for artificially aged parts.

Source	Force (L)			Displacement (D)		
	Mean of Square	F Value	*p* Value Prob > F	Mean of Square	F Value	*p* Value Prob > F
Model	0.035	105.203	<0.001	0.079	93.528	<0.001
R	0.087	133.613	<0.001	0.095	123.007	<0.001
α	0.039	100.763	<0.001	0.083	87.173	<0.001
K	0.073	167.224	<0.001	0.025	103.835	<0.001
R·α	1.17e−3	82.692	0.058	2.63e−3	94.122	0.002
R·K	1.98e−3	103.704	0.003	3.82e−3	73.464	0.521
K·α	2.39e−3	92.913	0.031	2.62e−3	83.174	0.003
R·K·α	1.23e−7	76.347	0.098	1.97e−3	67.258	0.796
R^2^	3.97e−3	93.173	0.143	3.81e−3	105.681	0.521
α^2^	2.09e−3	62.526	0.028	4.89e−3	78.687	0.231
K^2^	1.36e−3	73.231	0.099	2.11e−3	69.456	0.001
Residual	2.69e−3	69.523	-	3.29e−3	53.651	-

**Table 9:** Experiments used to confirm the mathematical model for force (L) and displacement (D) for artificially aged parts.

No. Exp.	Parameters			Force (L)			Displacement (D)		
	R (mm)	α°	K	Predicted Value	Actual Value	% Error	Predicted Value	Actual Value	% Error
1	0.1	1.0	0.2	129.005	131.105	1.601	1.726	1.825	5.424
2	0.4	2.0	0.4	269.545	254.512	−5.906	1.541	1.653	2.407
3	0.7	4.0	0.6	222.075	217.263	−2.214	1.428	1.372	−4.081
4	1.0	5.0	0.8	236.091	225.183	−4.844	1.408	1.396	−0.859
5	1.1	6.0	1.1	219.596	223.253	1.638	1.358	1.298	−4.622

**Table 10:** Reduced viscosity (*η*_red_) values for new and artificially aged parts.

Run Order	New Parts		Artificially Aged Parts	
	Reduced Viscosity (*η*_red_), cm^3^/g	Degradation, %	Reduced Viscosity (*η*_red_), cm^3^/g	Degradation, %
1	96.1	3.61	59.9	39.9
2	95.9	3.82	59.6	40.2
3	98.7	1.01	64.2	39.6
4	95.9	3.82	59.7	40.1
5	98.6	1.10	64.5	35.3
6	95.8	3.91	64.9	34.9
7	96.6	3.10	60.6	39.2
8	96.5	3.20	60.3	39.5
9	99.2	0.61	64.8	35.0
10	99.1	0.60	61.6	38.2
11	99.5	0.20	65.3	34.7
12	98.6	1.00	61.9	37.9
13	98.8	0.90	65.2	34.7
14	96.5	3.20	61.6	38.2
15	99.0	0.70	63.7	36.1
16	98.8	0.90	62.8	37.0
17	97.9	1.80	63.2	36.6
18	97.8	1.90	62.1	37.7
19	97.6	2.10	61.8	38.0
20	97.4	2.30	62.3	37.5

## Data Availability

The data presented in this study are available on request from the corresponding author.
